# A Dexterous Hand for Omnidirectional In-Hand Manipulation: Design, Analysis and Experimental Validation

**DOI:** 10.3390/biomimetics11030167

**Published:** 2026-03-02

**Authors:** Huaiyong Li, Changlong Ye, Rongdian Jia, Suyang Yu, Guanghong Tao

**Affiliations:** 1 School of Mechatronics Engineering, Shenyang Aerospace University, Shenyang 110136, China; 2 Key Laboratory of Rapid Development & Manufacturing Technology for Aircraft, Ministry of Education, Shenyang 110000, China; 3 School of Artificial Intelligence, Shenyang Aerospace University, Shenyang 110136, China

**Keywords:** dexterous hand, mecanum wheel, omnidirectional manipulation, kinematics

## Abstract

Traditional dexterous hands can readily grasp objects but face limitations in dexterous manipulation due to complex control systems and high actuation demands. This paper presents a novel dexterous hand designed to address these challenges. The hand consists of four fingers, each equipped with two mecanum wheels at the fingertips to allow for the omnidirectional manipulation of objects. Continuous rotation of the mecanum wheels enables unbounded motion of grasped objects without the need for finger gaiting. Object pose adjustment is achieved by controlling the rotation of mecanum wheels, thus significantly reducing operational complexity and enhancing manipulative agility. Furthermore, to address the control difficulty of multi-finger coordinated motion, a four-finger coupled mechanism is implemented, resulting in a dexterous hand with three degrees of freedom. Kinematic models of omnidirectional manipulation are established for typical geometric objects, including a flat plate, a cuboid, a sphere, and a cylinder. Simulations confirm the correctness of the kinematic models. Experimental results show that the hand can achieve omnidirectional manipulation of objects. Finally, the extended functionality of the dexterous hand is briefly presented, which allows it to be reconfigured into an omnidirectional mobile robot.

## 1. Introduction

As the end-effector of robots, dexterous hands play a pivotal role in facilitating interactions between robots and their environments. Their significance is increasingly recognized in fields such as industrial automation, healthcare, space exploration, and military applications. Notably, the advanced control and operational strategies for hybrid AC/DC networked microgrids proposed by Liang et al. [1] have further revealed that the unique operational flexibility of dexterous hands can serve as a critical enabler for enhancing energy security in hazardous or remote operational scenarios. Unlike simple grippers, which are often limited to single-object picking, fixed actions, and simplistic designs, dexterous hands can fully exploit their flexibility to grasp a wide range of objects and perform versatile operations, thereby addressing diverse operational demands [2,3]. C. Piazza et al. provided a comprehensive review of historical developments in robotic hand designs [4], showing a trend towards simplification.

Numerous researchers have attempted to replicate manipulative capabilities by mimicking the structural and functional characteristics of the human hand. Early examples of hands with in-hand manipulation capabilities include the Stanford/JPL hand from Stanford University and the Utah/MIT hand from the University of Utah and the Massachusetts Institute of Technology [5,6]. The BH-3 dexterous hand, developed by the Institute of Robotics at Beihang University, is modeled after the Stanford/JPL hand and incorporates three fingers with nine degrees of freedom, primarily serving as a platform for research on multi-fingered manipulation theories [7]. The UBhand series, developed by the University of Bologna in Italy, employs a modular design with elastic skeletal joints, artificial tendons distributed along the skeletal surface, and a flexible outer covering to enhance grasping stability [8,9,10,11]. The Metamorphic Hand, a collaborative effort between King’s College London and Tianjin University, introduces a reconfigurable palm based on a spherical five-bar linkage mechanism, significantly improving dexterity and versatility [12]. A biomimetic dexterous hand developed at the University of Washington replicates human hand characteristics using artificial joint capsules, ligaments, tendons, and an elastic pulley system, achieving highly flexible and reliable grasping capabilities [13]. This design has greatly advanced the understanding of human hand structure and function. Their high cost, coupled with complex sensing and control requirements, has limited their application beyond laboratory settings.

Many soft robotic hands with varying degrees of flexibility have been developed. Shintake et al. [14,15] proposed a spectrum of soft joint implementations, ranging from articulated structures that derive flexibility from elastic elements to fully flexible systems capable of continuous deformation into countless shapes. Soft joint designs are often inspired by the human musculoskeletal system, resulting in compliant systems with concentrated flexibility at the joints. Examples include the S Hand [16], Alpha Hand [17], Delft Cylinder Hand [18], Bionic Hand [19], FRH-4 Hand [20], and Keio Hand [21]. However, these designs frequently face challenges such as complex control mechanisms and reduced durability.

Some researchers have investigated a range of alternative dexterous hand designs. For instance, Xiaodong Jin et al. [22] designed two dexterous hands based on parallel finger structures, which exhibit high dexterity and load-carrying capacity. McCann et al. [23,24] enhanced the capabilities of a robotic gripper by integrating conveyor belts, enabling advanced in-hand manipulation techniques. Shenli Yuan et al. [25,26,27] developed two types of grippers: one utilizes articulated rollers at the fingertips, providing a manipulable surface for grasping and continuously rotating objects without complex finger repositioning; the other incorporates spherical wheels at the fingertips, offering two degrees of freedom: rotational motion for object reorientation and continuous rolling for manipulation. However, these systems are nonholonomic, meaning that the grasped object cannot be moved instantaneously in an arbitrary direction and may require a series of motions to achieve a desired pose.

The main contributions of this study can be summarized as follows:

(1) It developed a novel dexterous hand.
(2) It designed a four-finger coupled mechanism that significantly reduces the control complexity for hand reconfiguration.
(3) It proposes an innovative fingertip design utilizing mecanum wheels, enabling omnidirectional manipulation solely through wheel speed control without complex finger gait planning, thereby dramatically simplifying the control challenge.
(4) It achieved the grasping of parts, pose adjustment, and subsequent assembly.

The structure of this paper is as follows. Section 2 describes the design of the dexterous hand. Section 3 develops the mathematical models. Section 4 verifies the correctness of the models. Section 5 reports experimental results. Section 6 briefly introduces the extensible functionality of the dexterous hand. Section 7 concludes the paper and discusses future work.

## 2. Design

### 2.1. Bionic Analysis

**Figure 1:**
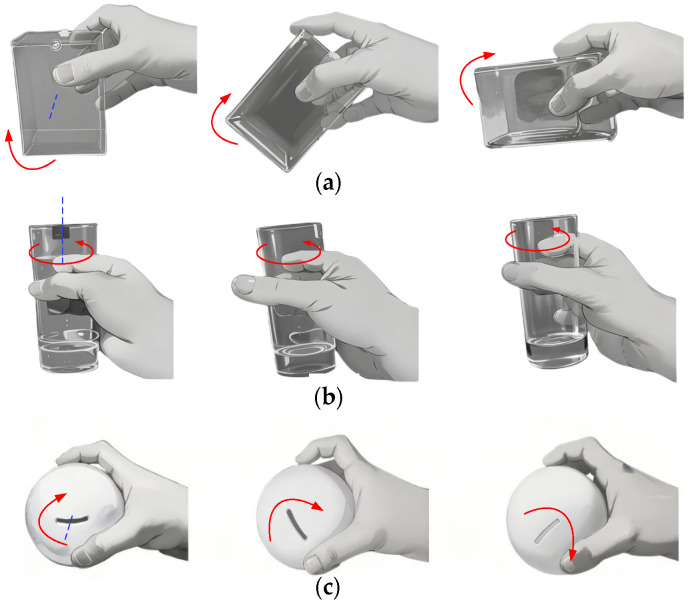
Human hand manipulation: (**a**) Rotating a cuboid; (**b**) Rotating a cylinder; (**c**) Rotating a sphere. The red arrow indicates the direction of rotation of the object.

The human hand is a remarkable biomechanical system, equipped with multiple joints and degrees of freedom that enable a wide range of motion. This versatility allows the hand to grasp objects of varying shapes and sizes and perform complex tasks with precision and adaptability. Its design, characterized by features such as opposable thumbs and finely tuned muscle-tendon coordination, makes it one of the most dexterous tools in nature. Owing to its exceptional flexibility and functional versatility, the structure and kinematic principles of the human hand provide critical insights for the design of advanced dexterous hands. Understanding the movement and environmental interactions of the human hand can significantly enhance the development of dexterous hands capable of performing tasks requiring high levels of dexterity. Figure 1 illustrates the human hand manipulating a cuboid, a cylinder, and a sphere.

During object manipulation, the human hand typically relies on complex finger gaits, which involve cyclic processes of contact, detachment, and re-contact between the fingers and the object. Currently, most dexterous hand designs still emulate the structure of the human hand. When performing in-hand manipulation tasks, the frequent switching of finger gaits significantly increases the complexity and difficulty of control.

### 2.2. Concept Design

**Figure 2:**
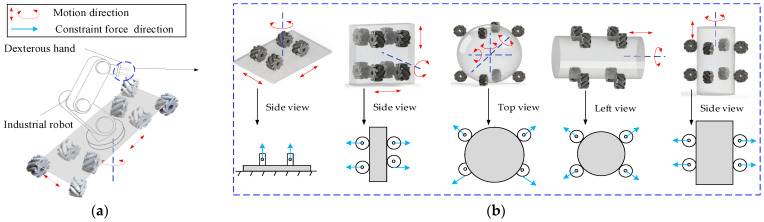
Concept design: (**a**) Omnidirectional mobile robot; (**b**) Omnidirectional manipulation.

The design was inspired by mecanum wheels, which are characterized by their omnidirectional movement. On one hand, arranging multiple mecanum wheels in different configurations enables arbitrary planar motion, as illustrated in Figure 2a; on the other hand, by employing mecanum wheels as the fingertips of a dexterous hand, omnidirectional motion of in-hand objects can be achieved through the combined deformation of its four fingers, as shown in Figure 2b. Generally, objects can be considered as combinations of basic geometric primitives, such as cylinders, cuboids, and spheres. Therefore, for the purpose of grasp categorization and analysis, these three fundamental types are representative of most objects encountered in practice [28].

**Figure 3:**
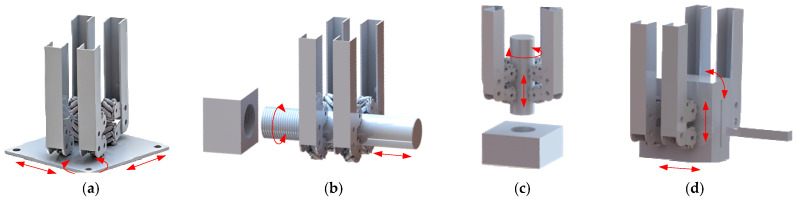
Applications: (**a**) Manipulating a plate-shaped part; (**b**) Manipulating a threaded shaft; (**c**) Manipulating a smooth shaft; (**d**) Manipulating a cuboid-shaped part. The red arrow indicates the direction of rotation of the object.

The mecanum-wheel-based dexterous hand not only enhances the operational flexibility of in-hand objects but also realizes omnidirectional manipulation solely by controlling the rotational speeds of the mecanum wheels. This approach eliminates the complex finger gaits required for object manipulation in traditional dexterous hands, thereby significantly reducing control difficulty. To enhance grasp stability and enable effective omnidirectional manipulation, eight mecanum wheels are utilized. The reconfiguration of mecanum wheel arrangements is accomplished through a pose adjustment mechanism. In practical applications, the dexterous hand can be integrated via an industrial robot onto an omnidirectional mobile robot reconfigured from this same hand, as illustrated in Figure 2a. This integrated system achieves both omnidirectional mobility and omnidirectional manipulation of grasped objects, making it applicable for tasks such as part handling and assembly in industrial automation. In the figures, the red arrows indicate the motion direction of the objects, while the blue arrows represent the direction of the constraint forces exerted by the wheels when contacting the object in different operational states. Figure 3 illustrates typical applications of this dexterous hand. It enables pose adjustment of grasped objects without employing finger gaits, and then proceeds to complete subsequent tasks.

### 2.3. Scheme Design

**Figure 4:**
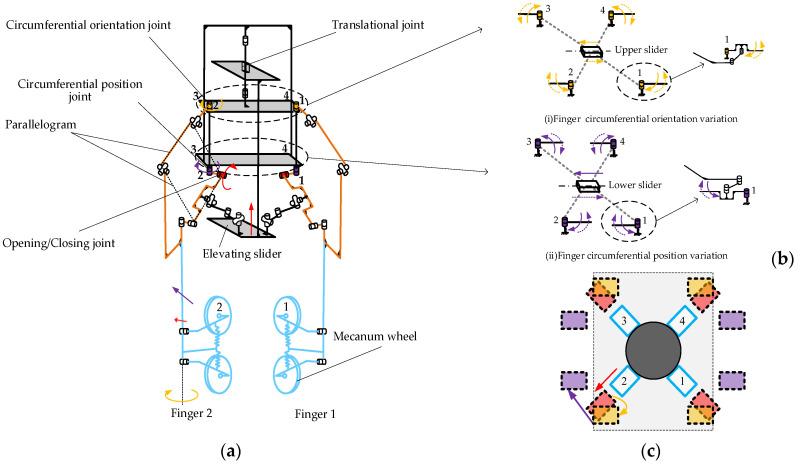
Schematic diagram of mechanism motion: (**a**) Overall mechanism motion; (**b**) Four-finger linkage mechanism; (**c**) Fingers’ three-degree-of-freedom motion. The roman numerals correspond to the respective fingers; all colored arrows indicate the direction of motion.

Based on the structural characteristics of the human hand and the kinematic principle of mecanum wheels, we designed a dexterous hand with enhanced maneuverability yet a reduced number of degrees of freedom. Figure 4a presents a schematic of the dexterous hand (note: the 3rd and 4th fingers are omitted for clarity). To maintain a constant pointing direction of the finger during deformation, which is crucial for stable grasping and effective object manipulation, we implemented the spatial parallelogram mechanism. This mechanism comprises six moving components, two fourth-class kinematic pairs and five fifth-class kinematic pairs, resulting in a system with three degrees of freedom, as expressed in Equation (1).

(1) $$F=6n-\sum_{i=1}^{5}ip_{i}=6\times 6-\left(4\times 2+5\times 5\right)=3$$

where *n* is the number of movable components; *i* is the number of constraints of the *i*-level kinematic pair; *p_i_* is the number of *i*-level kinematic pairs.

Individually controlling the four fingers would increase both the degrees of freedom and the control complexity. To address this, a four-finger linkage control scheme is employed. As shown in Figure 4a, the vertical motion of the elevating slider drives four opening/closing joints to rotate simultaneously with the same angular displacement. As shown in Figure 4b, the upper slider drives four circumferential orientation joints to rotate with the same angular displacement, while the lower slider drives four circumferential position joints to rotate with the same angular displacement. The vertical motion of the elevating slider controls the opening and closing deformation of the dexterous hand. The motions of upper and lower sliders control the circumferential deformation of the fingers: one regulates the finger orientation, and the other adjusts the finger position. As shown in the top view of Figure 4c, the dexterous hand first grasps a cylinder. It then performs three sequential steps: opening, circumferential orientation adjustment, and circumferential position adjustment, thus reconfiguring itself into a configuration suitable for grasping a cuboid.

### 2.4. Mechanical Structure

**Figure 5:**
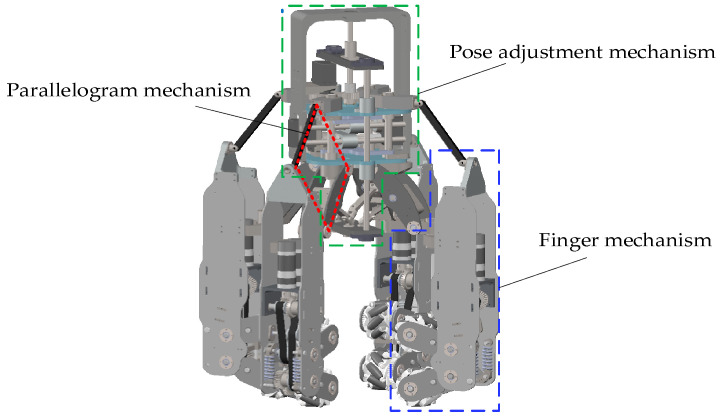
Three-dimensional model of the dexterous hand.

As shown in Figure 5, the three-dimensional model of the dexterous hand comprises four finger mechanisms, four sets of spatial parallelogram mechanisms, and a pose adjustment mechanism.

#### 2.4.1. Finger Mechanism

**Figure 6:**
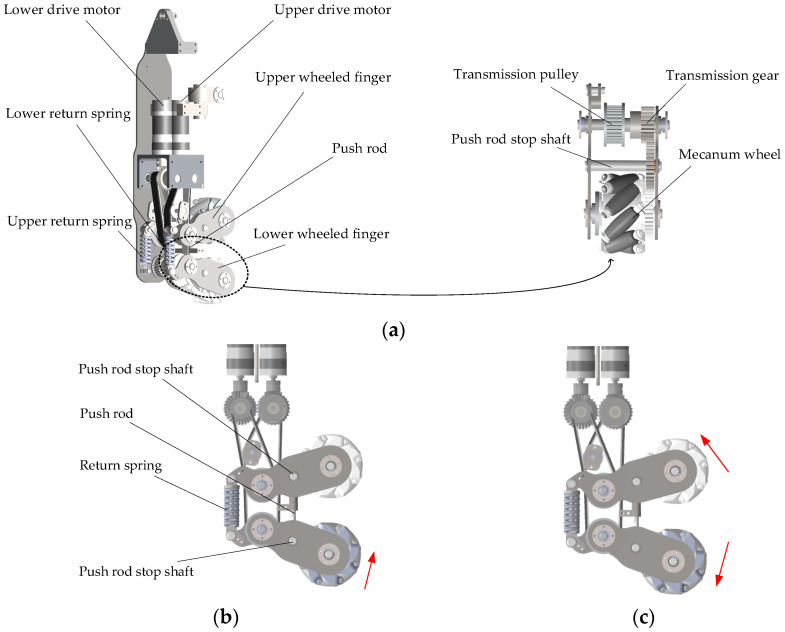
Finger: (**a**) Finger mechanism; (**b**) Four-wheeled mode; (**c**) Eight-wheeled mode. The red arrow indicates the motion direction of wheeled finger.

The finger mechanism is depicted in Figure 6a, each finger consists of two wheel-finger units connected by pivot joints, with each unit equipped with an actively driven mecanum wheel (rollers made of ABS engineering plastic). To enable effective and flexible object manipulation, the arrangement of the eight mecanum wheels across the four fingers follows specific principles. Specifically, the two mecanum wheels on each finger have rollers inclined in opposite directions, while adjacent mecanum wheels between fingers also maintain opposite roller inclinations. This configuration not only enables force-closure grasping but also permits the application of primary torque to the grasped object, allowing for full-range motions within a multi-degree-of-freedom space.

The power transmission system delivers torque to the mecanum wheels through conical gears, synchronous pulleys, and gears driven by dual motors. The two synchronous belts are of different lengths, and specifically, an idler pulley is installed at the longer synchronous belt to maintain optimal tension. Through adjustment of the motor base position, both synchronous belts can be simultaneously tensioned, thus ensuring proper power transmission.

Each wheeled finger is connected to the finger frame via a spring-damping system to achieve a certain degree of compliance during object grasping. The operational states are shown in Figure 6. In four-wheeled mode, only the lower wheeled fingers are engaged. Upon object contact, the lower fingers swing counterclockwise due to combined normal force and sliding friction (Figure 6b). They then push the upper wheeled finger structure counterclockwise via a push rod mechanism. Throughout this, both the push rod return spring and upper finger return spring stay compressed, maintaining constraint force during in-hand manipulation. In eight-wheeled mode, all wheeled fingers are engaged. Upon contact, the upper and lower fingers swing in opposite directions due to contact forces (Figure 6c). Both upper and lower return springs remain compressed, sustaining the necessary constraint force for in-hand manipulation.

#### 2.4.2. Spatial Parallelogram Mechanism

During the transformation process of the dexterous hand, the poses of the fingers change while the pointing directions of the four fingers remain unchanged. To achieve this functionality, a three-dimensional variation in the planar parallelogram mechanism, known as the spatial parallelogram mechanism, is adopted between the pose adjustment mechanism and the finger.

**Figure 7:**
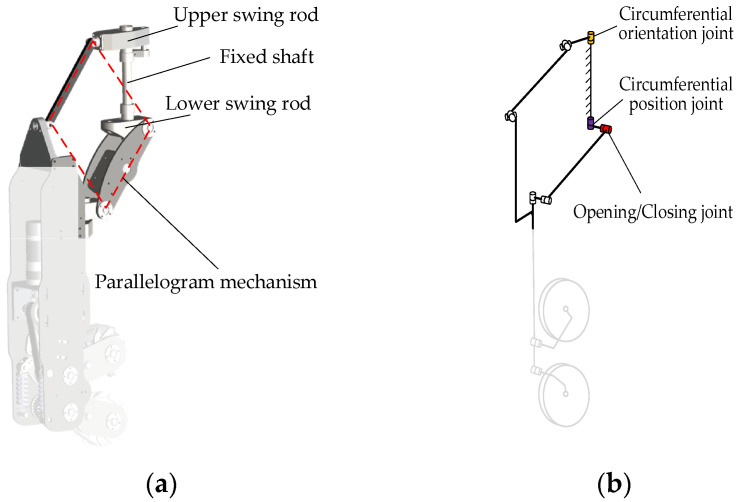
Spatial parallelogram mechanism: (**a**) Three-dimensional model; (**b**) Schematic diagram.

Figure 7a illustrates the three-dimensional model of the spatial parallelogram, while Figure 7b shows the schematic diagram of the mechanism. The fixed shaft is mounted on the frame and is rigidly connected to it. Both the circumferential orientation and position joints are located at the ends of the fixed shaft and function as the power input for the spatial parallelogram mechanism. Specifically, the circumferential orientation joint is connected to the output shaft through a cross universal joint-link-cross universal joint configuration, whereas the circumferential position joint is connected to the output shaft via a swing joint-link-swing joint configuration.

#### 2.4.3. Pose Adjustment Mechanism

**Figure 8:**
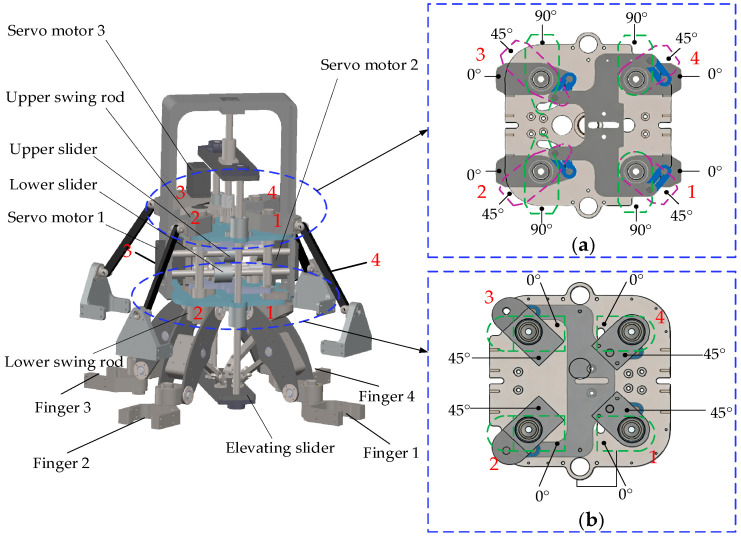
Pose adjustment mechanism: (**a**) Position of upper swing rod joints; (**b**) Position of lower swing rod joints. The roman numerals correspond to the respective fingers.

The pose adjustment mechanism, as shown in Figure 8, comprises a lead screw, transmission gears, upper/lower sliders, swing rod joints, and an elevating slider. Connected to four fingers through spatial parallelogram mechanisms, it controls both opening/closing and circumferential deformation.

In opening/closing deformation, the elevating slider is linked to the fingers. Servo motor 3 drives its vertical movement via a gear and lead screw system. In circumferential deformation, servo motors 1 and 2 are coupled to two lead screws, each independently driving an upper and a lower sliding rod mechanism. The upper mechanism controls finger orientation, while the lower regulates finger position.

Each sliding rod mechanism comprises four swing rods. Figure 8a shows the upper swing rods operating at 0–90° phase angles; Figure 8b shows the lower swing rods at 0–45° phase angles. The reciprocating motion of the sliders drives their respective swing rods through equal angular displacements. These rods connect to the fingers via linkages to achieve circumferential deformation. During the grasping of objects with varied shapes, the phase angles of the swing rod joints are listed in Table 1.

## 3. Kinematics

### 3.1. Body Kinematics

**Figure 9:**
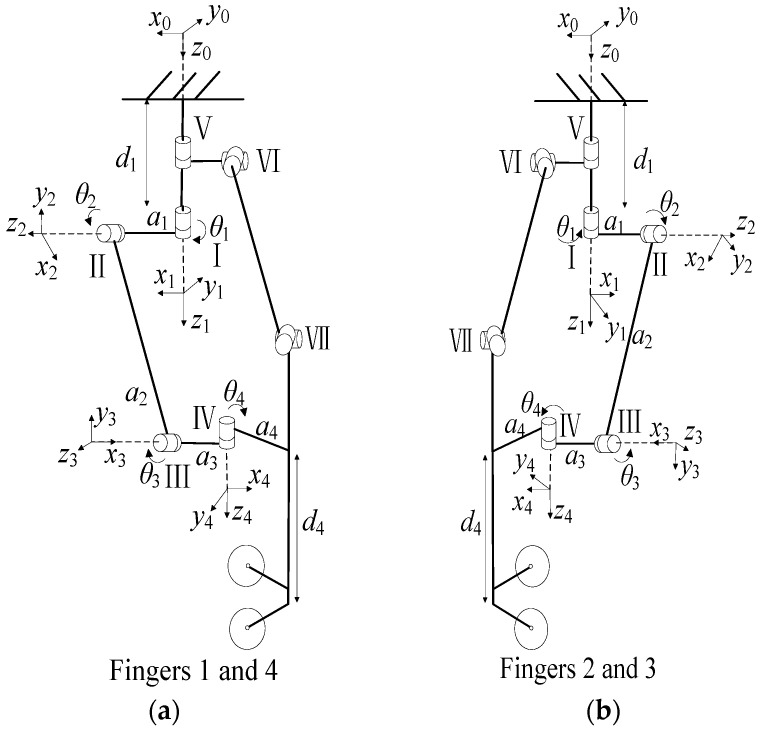
Finger coordinate systems: (**a**) Fingers 1 and 4; (**b**) Fingers 2 and 3. The black arrows indicate the direction of joint rotation.

The coordinate systems are established as illustrated in Figure 9, with the corresponding Denavit-Hartenberg (D-H) parameters for each finger link coordinate system provided in Table 2.

T10=cosθ1−sinθ10−a0sinθ1cosθ10−b0001c00001 (Finger 1)T10=cosθ1−sinθ10−a0sinθ1cosθ10b0001c00001 (Finger 4)T21=cosθ2−sinθ20a100−10sinθ2cosθ2000001T32=cosθ3−sinθ30a2sinθ3cosθ30000100001T43=cosθ4−sinθ40a300−10sinθ4cosθ4000001T54=100a40100001d50001

By multiplying the above transformation matrices, the kinematic models for Finger 1 and Finger 4 are derived as follows:

(2) T50=T10T21T32T43T54=nxoxaxpxnyoyaypynzozazpz0001

T′10=cosθ1−sinθ10a0sinθ1cosθ10−b0001c00001 (Finger 2) T′10=cosθ1−sinθ10a0sinθ1cosθ10b0001c00001 (Finger 3)T′21=cosθ2−sinθ20a10010−sinθ2−cosθ2000001T′32=cosθ3−sinθ30a2sinθ3cosθ30000100001T′43=cosθ4−sinθ40a30010−sinθ4−cosθ4000001T′54=100a40100001d50001

By multiplying the above transformation matrices, the kinematic models for Finger 2 and Finger 3 are derived as follows:

(3) T′50=T′10T′21T′32T′43T′54=nxoxaxpxnyoyaypynzozazpz0001

### 3.2. Manipulation Kinematics

The manipulation kinematics is expressed as the mapping relationship between the velocities of the mecanum wheels and the velocity of the target object. The kinematic equation can be formulated as:

(4) $$r\dot{\varphi_{i}}\;=\;J\dot{\psi }$$

where *r* denotes the radius of the mecanum wheel, $\dot{\varphi_{i}}$ represents the angular velocity vector of the mecanum wheels, $\dot{\psi }$ denotes the target object’s generalized velocity vector, and ***J*** represents the Jacobian matrix.

**Figure 10:**
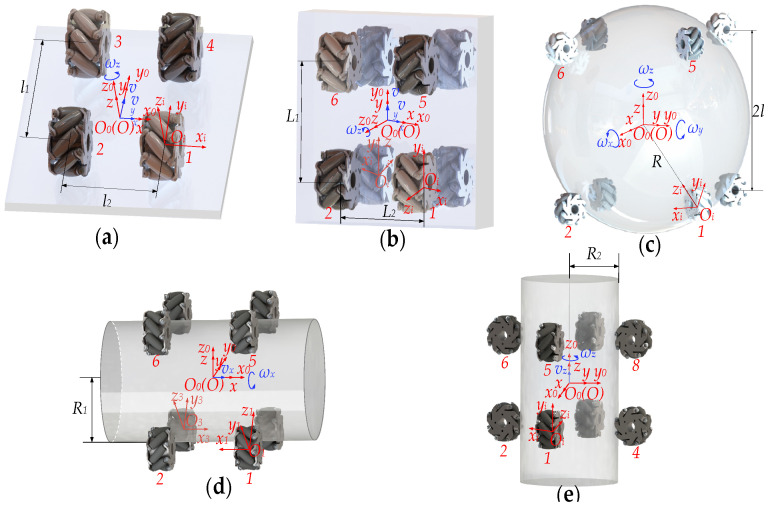
Coordinate systems for object manipulation: (**a**) Flat plate; (**b**) Cuboid; (**c**) Sphere; (**d**) Horizontal cylinder; (**e**) Vertical cylinder. The blue arrow indicates the direction of motion of the object.

The coordinate systems are established as shown in Figure 10, where *O*_0_-*x*_0_*y*_0_*z*_0_ denotes the global coordinate system, *O-xyz* denotes the object coordinate system, and *O_i_-x_i_y_i_z_i_* denotes the mecanum wheel coordinate system.

As shown in Figure 10a, when manipulating a flat plate, four mecanum wheels integrated at the end-effector to maintain firm contact with the flat plate. Through coordinated actuation of these omnidirectional wheels, the system achieves full three-degree-of-freedom planar motion of the manipulated object. The Jacobian matrix is given by:

(5) $$J_{f}=\left[\begin{array}{ccc} 1 & 1 & y-x+m\\ -1 & 1 & -y-x-m\\ 1 & 1 & y-x-m\\ -1 & 1 & -y-x+m \end{array}\right]$$

where (*x*, *y*) represents the coordinates of the flat plate’s reference point, $m=\frac{l_{1}+l_{2}}{2}$.

As shown in Figure 10b, when manipulating a cuboid, the rotational motion of eight mecanum wheels enables the cuboid to achieve three-degree-of-freedom movement. The Jacobian matrix is as follows:

(6) $$J_{c}=\left[\begin{array}{ccc} 1 & 1 & y-x+n\\ -1 & 1 & -y-x-n\\ -1 & 1 & -y-x-n\\ 1 & 1 & y-x+n\\ -1 & 1 & -y-x+n\\ 1 & 1 & y-x-n\\ 1 & 1 & y-x-n\\ -1 & 1 & -y-x+n \end{array}\right]$$

where (*x*, *y*) represents the coordinates of the cuboid’s reference point, $n=\frac{L_{1}+L_{2}}{2}$.

As illustrated in Figure 10c, when manipulating a sphere, the eight Mecanum wheels enable it to rotate about any axis passing through its center. The Jacobian matrix is as follows:

(7) $$J_{s}=\left[\begin{array}{ccc} -a & b & c\\ a & b & c\\ a & -b & c\\ -a & -b & c\\ -a & b & c\\ a & b & c\\ a & -b & c\\ -a & -b & c \end{array}\right]$$

where $a=\frac{\sqrt{2}R(R+r+l)}{2\left(R+r\right)}$, $b=\frac{\sqrt{2}R(R+r-l)}{2\left(R+r\right)}$, $c=\frac{R\sqrt{{\left(R+r\right)}^{2}-l^{2}}}{R+r}$.

As shown in Figure 10d,e, when manipulating a horizontal cylinder and a vertical cylinder, the rotation of the wheels drives the cylinder to perform both translation and rotation relative to its own axis. The Jacobian matrices are as follows:

(8) $$J_{1}=\left[\begin{array}{cc} -1 & R_{1}\\ 1 & R_{1}\\ 1 & {-R}_{1}\\ -1 & -R_{1}\\ 1 & R_{1}\\ -1 & R_{1}\\ -1 & {-R}_{1}\\ 1 & {-R}_{1} \end{array}\right]\;J_{2}=\left[\begin{array}{cc} -1 & {-R}_{2}\\ -1 & R_{2}\\ -1 & -R_{2}\\ -1 & R_{2}\\ -1 & R_{2}\\ -1 & {-R}_{2}\\ -1 & R_{2}\\ -1 & {-R}_{2} \end{array}\right]$$

where *R*_1_ and *R*_2_ represent the circular cross-sectional radius of the cylinder.

## 4. Simulation

In order to verify the correctness of the established kinematic model, this section described the performed simulations. The verification process follows three key steps: First, the desired velocity of the target object is specified as input to the kinematic equation, from which the required mecanum wheel velocities are derived. Subsequently, these calculated wheel velocities are imported into a virtual prototype environment. Finally, the simulated velocity profiles of the target object are obtained and compared with theoretical predictions to establish motion consistency.

**Figure 11:**
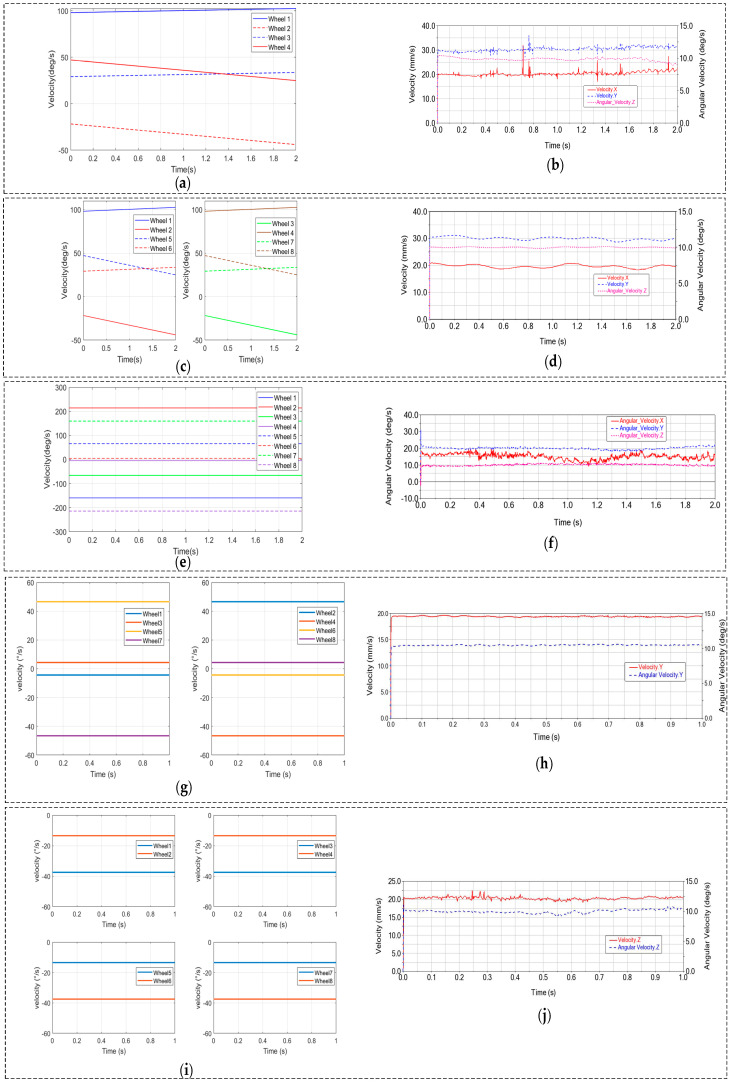
Simulation results: (**a**) Wheel velocities i; (**b**) Flat plate velocities; (**c**) Wheel velocities ii; (**d**) Cuboid velocities; (**e**) Wheel velocities iii; (**f**) Sphere velocities; (**g**) Wheel velocities iv; (**h**) Horizontal cylinder velocities; (**i**) Wheel velocities v; (**j**) Vertical cylinder velocities.

For the flat plate and cuboid, translational velocities of 20 mm/s (x_0_-axis) and 30 mm/s (y_0_-axis), along with a rotational velocity of 10 deg/s (z_0_-axis), were commanded. The required rotational velocities for the four (plate) or eight (cuboid) mecanum wheels, calculated via the kinematic equations (Figure 11a,c), were input into the virtual prototype. The resulting simulated velocity profiles Figure 11b,d) successfully replicated the prescribed 3-DOF composite motions, confirming the system’s capability for synchronous translation and rotation.

For the sphere, rotational velocities of 10 deg/s (x_0_), 15 deg/s (y_0_), and 20 deg/s (z_0_) were set. The corresponding wheel velocities derived from kinematics (Figure 11e) were applied, and the simulation (Figure 11f) accurately reproduced the 3-DOF rotational motion, demonstrating synchronous rotation about all three axes.

During cylinder manipulation, a linear velocity of 20 mm/s and an angular velocity of 10 deg/s about its axis were set. Applying the wheel velocities calculated from the kinematic equations (Figure 11g,i) to the virtual prototype yielded simulated profiles (Figure 11h,j) that successfully matched the desired cylinder motions.

## 5. Experiment

**Figure 12:**
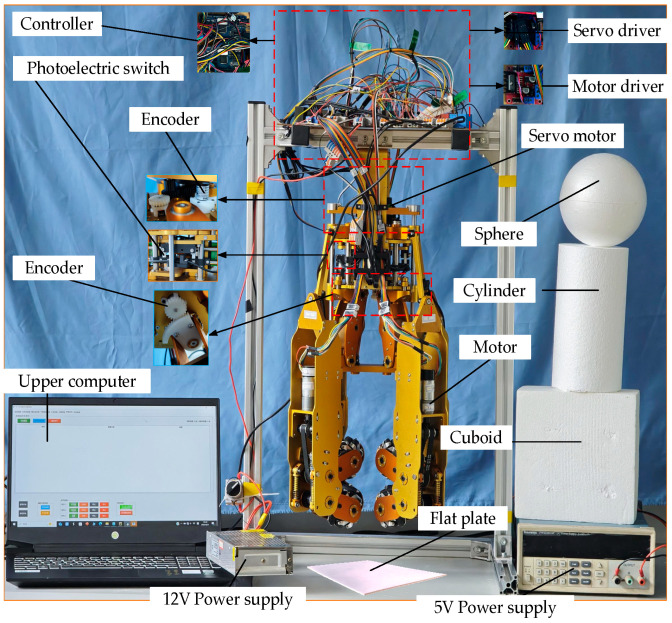
Experimental platform.

To validate the omnidirectional manipulation capability of the dexterous hand, this study conducted grasping and manipulation experiments on several typical objects, including a flat plate, a cuboid, a sphere, and a cylinder. As shown in Figure 12, the experimental platform mainly consists of the dexterous hand body, power supply, upper computer, controller, driver, encoder and servo motor. The key technical parameters of the prototype are summarized in Table 3.

### 5.1. Objects Grasping

**Figure 13:**
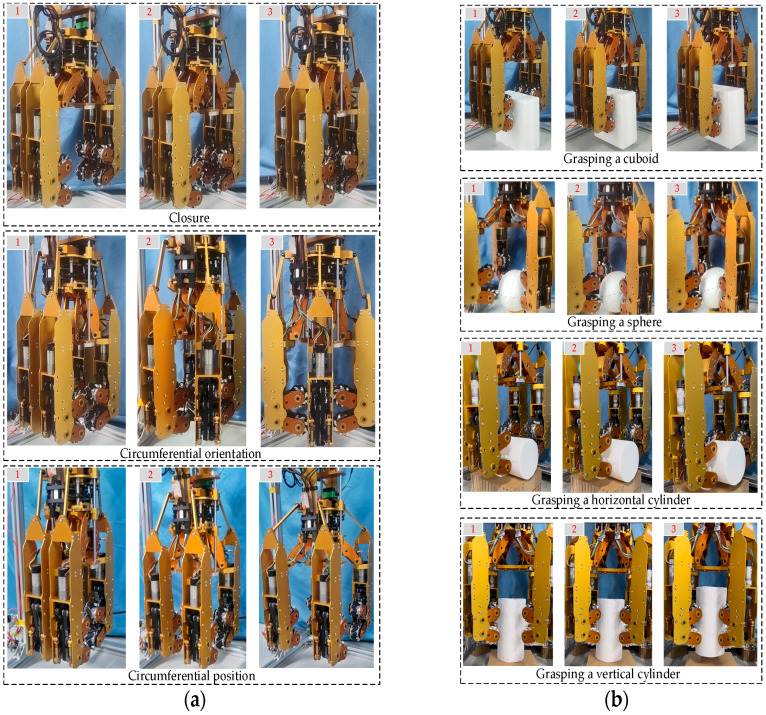
Deformation and grasping: (**a**) Deformation process; (**b**) Objects grasping.

Figure 13a shows the deformation processes corresponding to the three degrees of freedom of the dexterous hand. Figure 13b demonstrates the hand’s ability to successfully grasp geometrically distinct objects, including a cuboid, a sphere, and a cylinder, by effectively synthesizing these degrees of freedom into a unified composite deformation strategy, thereby validating its adaptability across diverse shapes.

### 5.2. Objects Manipulating

In this section, based on the kinematic model, the desired angular velocities of the mecanum wheels are derived from the specified object velocity. An open-loop control scheme is employed to validate the fundamental omnidirectional manipulation capabilities of the system.

**Figure 14:**
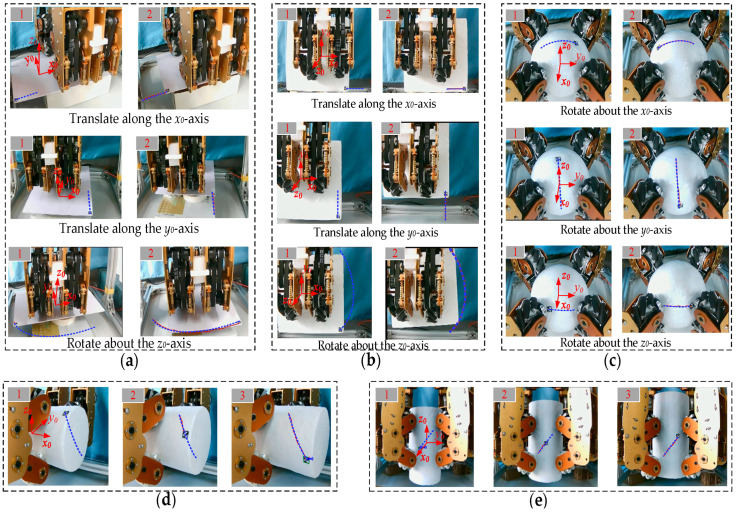
Omnidirectional manipulations: (**a**) Manipulating a flat plate; (**b**) Manipulating a cuboid; (**c**) Manipulating a sphere; (**d**) Manipulating a horizontal cylinder; (**e**) Manipulating a vertical cylinder.

As shown in Figure 14a, through the four-wheel actuation system, the flat plate achieves omnidirectional motion within its operational degrees of freedom in the O-x_0_y_0_ plane. The sequential diagrams specifically capture the plate’s performance during a 0.1 m translation along the x_0_-axis, a 0.1 m translation along the y_0_-axis, and a π/2 rotation about the z_0_-axis, where the desired and actual trajectories are denoted by blue and red lines, respectively.

Figure 14b demonstrates the dexterous hand manipulating the cuboid. Through eight-wheel actuation system, the cuboid achieves omnidirectional planar motion within its operational degrees of freedom in the O-x_0_y_0_ plane. The sequential diagrams specifically capture the cuboid’s performance during a 0.1 m translation along the x_0_-axis, a 0.1 m translation along the y_0_-axis, and a π/2 rotation about the z_0_-axis.

Figure 14c shows the movement of the sphere. The sphere maintains uninterrupted contact with the eight mecanum wheels during operation. The sequential diagrams specifically capture the sphere’s performance during a π/3 rotation about the x_0_-axis, a π/2 rotation about the y_0_-axis, and a π/6 rotation about the z_0_-axis.

As shown in Figure 14d, the dexterous hand translates the horizontal cylinder by 0.1 m along the x_0_-axis while rotating it by π/3; Figure 14e illustrates the translation of the vertical cylinder by 0.1 m along the z_0_-axis with a simultaneous rotation of π/6.

**Figure 15:**
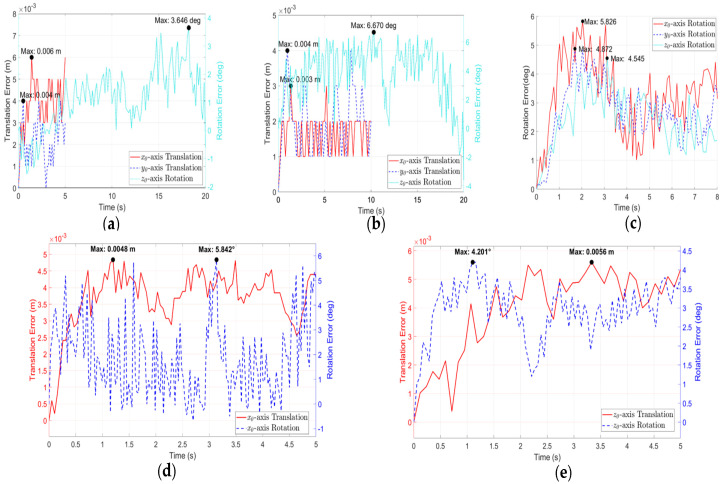
Trajectory tracking errors: (**a**) Flat plate; (**b**) Cuboid; (**c**) Sphere; (**d**) Horizontal cylinder; (**e**) Vertical cylinder.

As shown in Figure 15, the maximum trajectory tracking errors during the flat plate motion are 0.006 m, 0.004 m, and 3.646°. For the cuboid motion, the maximum trajectory tracking errors are 0.003 m, 0.004 m, and 6.670°. During the sphere motion, the maximum trajectory tracking errors are 4.872°, 5.826°, and 4.545°. During the horizontal cylinder motion, the maximum trajectory tracking errors are 0.0048 m and 5.842°. During the vertical cylinder motion, the maximum trajectory tracking errors are 0.0056 m and 4.201°.

These errors can be attributed to a combination of factors, including: (a) the inherent limitations of open-loop control; (b) possible slight vibration and slippage at the wheel-object interface, potentially induced by the discontinuous structure of the mecanum wheel rollers; and (c) unmodeled dynamics and mechanical clearances within the system. To mitigate these errors and enhance manipulation precision, future work will focus on developing closed-loop control strategies that integrate dynamic models with visual feedback.

**Figure 16:**
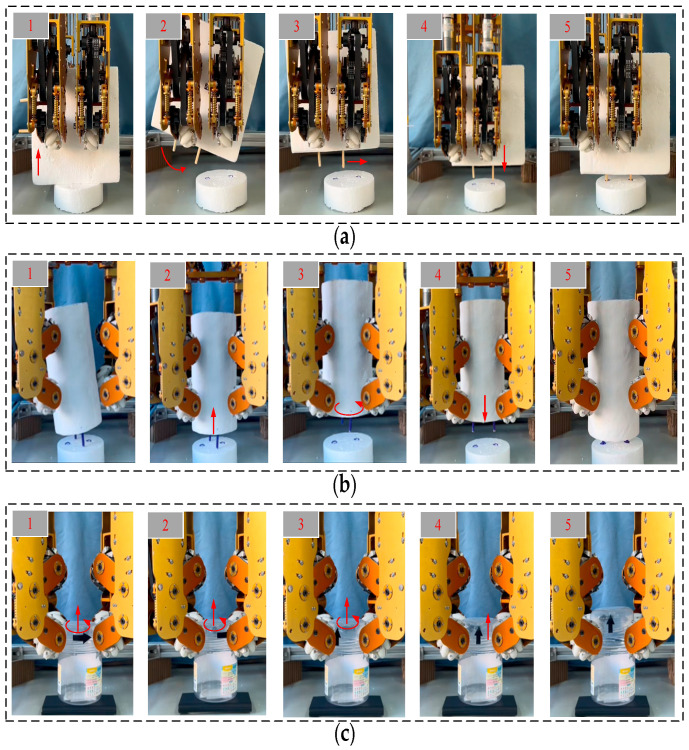
Simulated application: (**a**) Insert plug into socket; (**b**) Shaft-hole assembly; (**c**) Screw on a cap. The red arrow indicates the direction of motion of the object.

Figure 16a,b depict the sequential operations of grasping, in-hand pose adjustment, and assembly for a simulated workpiece or a power plug. Figure 16c depicts the twisting operation of a plastic bottle cap. The experimental results confirm that the proposed dexterous hand exhibits robust grasping stability and flexible manipulation capabilities for geometrically distinct objects.

## 6. Extended Functionality

**Figure 17:**
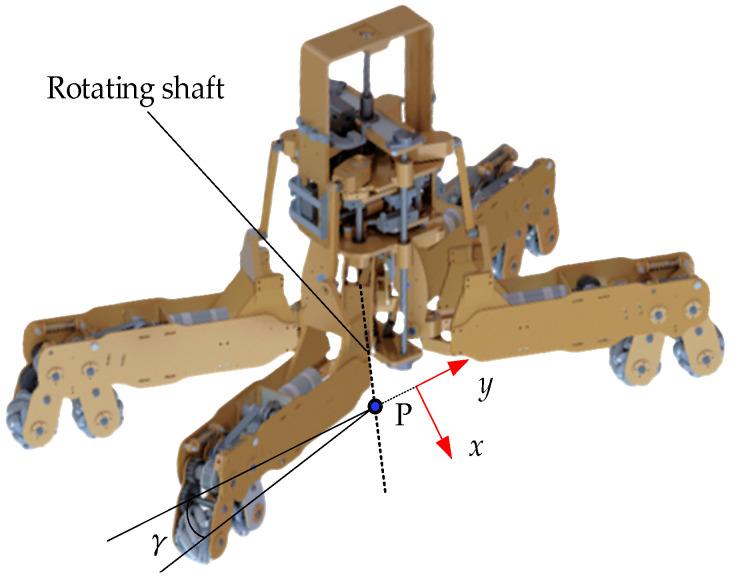
Omnidirectional mobile robot.

**Figure 18:**
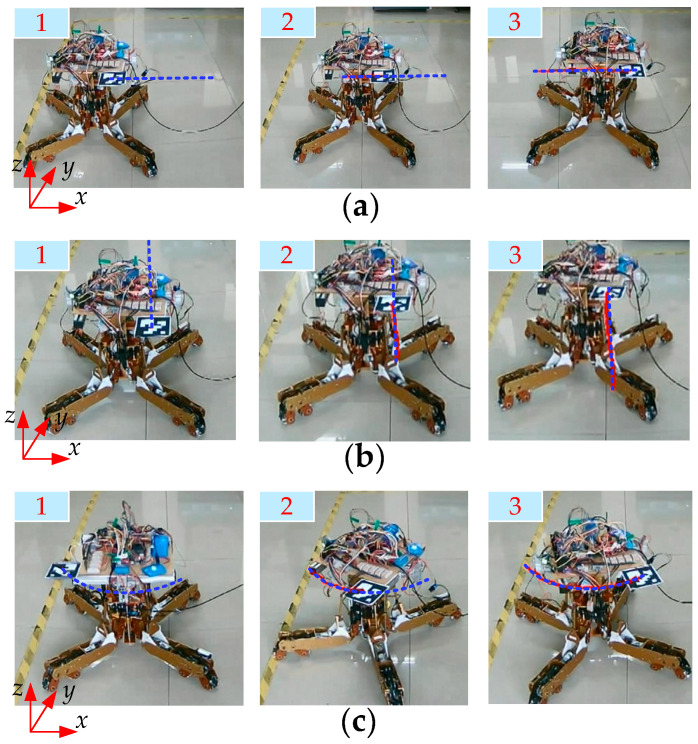
Omnidirectional motion: (**a**) Translation along the x-axis; (**b**) Translation along the y-axis; (**c**) Rotation about the z-axis.

Figure 17 shows the extended functionality of the dexterous hand, which can be reconfigured into an omnidirectional mobile robot, enabling three degrees of freedom motion on a plane and allowing for flexible movement in confined spaces. Point P in the figure denotes the intersection of the rotating shaft and the line connecting the centers of the two mecanum wheel hubs. The offset angle γ (γ ∈ [0°, 90°]) is defined as the angle between the line connecting the centers of the two mecanum wheels and the y-axis. Figure 18 illustrates the omnidirectional mobility of the robot (γ = 45°). The desired and actual trajectories are denoted by blue and red lines, respectively.

## 7. Conclusions and Future Work

This study presents a novel dexterous hand capable of reliable grasping and omnidirectional manipulation through a simplified control framework. Compared to traditional dexterous hands, the proposed system achieves two key advancements: dexterity enhancement is realized through actively driven mecanum wheels integrated as fingertips, which enable omnidirectional object motion without requiring finger detachment. This innovation effectively eliminates the inefficiencies inherent in traditional gait-based approaches; furthermore, control simplification is accomplished via a three-degree-of-freedom mechanism that synchronizes finger reorientation with radial displacement, thereby reducing actuation complexity from the multiple degrees of freedom requirements of anthropomorphic designs to three centralized degrees of freedom. In addition, the dexterous hand can be reconfigured into an omnidirectional mobile robot capable of arbitrary motion with three degrees of freedom in the plane.

Future work will focus on developing dynamic models incorporating contact mechanics. Advanced control strategies including hybrid force-motion control, adaptive sliding mode control, and uncertainty compensation will be investigated to enhance manipulation precision. Systematic evaluation of irregular object manipulation and environmental interaction effects will further validate the system’s practical applicability.

## Figures and Tables

**Table 1:** Phase angles of the swing rod joints.

Object	Upper Swing Rod Phase Angle	Lower Swing Rod Phase Angle
Flat plate	0°	0°/45°
Cuboid	0°	0°/45°
Sphere	45°	45°
Horizontal cylinder	0°	45°
Vertical cylinder	45°	45°

**Table 2:** D-H parameters of finger k.

i	$\alpha_{(i-1)1}$	$\alpha_{(i-1)2}$	$\alpha_{(i-1)3}$	$\alpha_{(i-1)4}$	$a_{(i-1)k}$	$d_{\mathrm{ik}}$	$\theta_{i1}$	$\theta_{i2}$	$\theta_{i3}$	$\theta_{i4}$
1	0	0	0	0	0	0	0°~45°	0°~−45°	180°~225°	−180°~−225°
2	90°	90°	−90°	−90°	*a*_1_	0	0°~90°	0°~−90°	0°~90°	0°~−90°
3	0	0	0	0	*a*_2_	0	75°~45°	75°~45°	−75°~−45°	−75°~−45°
4	90°	90°	−90°	−90°	*a*_3_	0	105°~135°	105°~135°	−105°~−135°	−105°~−135°
5	0	0	0	0	*a*_4_	*d*_5_	0	0	0	0

**Table 3:** Technical parameters of the prototype.

Parameter	Value (Unit)
Single finger	1.60 kg
Pose adjustment mechanism	4.40 kg
Total mass	12.00 kg
Retracted dimensions	0.141 m × 0.278 m × 0.593 m
Gripping thickness range	0.020 m–0.150 m
Grasping diameter	ϕ 0.070 m–ϕ 0.180 m

## Data Availability

The original data contributions presented in the study are included in the article, further inquiries can be directed to the corresponding author.
